# *trans*-Bis(ethyl­enediamine-κ^2^ *N*,*N*′)bis­(6-methyl-2,2,4-trioxo-3,4-dihydro-1,2λ^6^,3-oxathia­zin-3-ido-κ*N*)copper(II)

**DOI:** 10.1107/S1600536811051658

**Published:** 2011-12-14

**Authors:** Necmi Dege, Güneş Demirtaş, Hasan Içbudak

**Affiliations:** a Ondokuz Mayıs University, Arts and Sciences Faculty, Department of Physics, 55139 Samsun, Turkey; b Ondokuz Mayıs University, Arts and Sciences Faculty, Department of Chemistry, 55139 Samsun, Turkey

## Abstract

In the crystal structure of the title compound, [Cu(C_4_H_4_NO_4_S)_2_(C_2_H_8_N_2_)_2_], the Cu^2+^ ion resides on a centre of symmetry. The environment of Cu^2+^ ion is a distorted octa­hedron. The axial bond lengths between the Cu^II^ ion and the N atoms are considerably longer than the equatorial bond distances between the Cu^II^ ion and the N atoms of the ethyl­enediamine ligand as a consequence of the Jahn–Teller effect. The mol­ecular conformation is stabilized by intra­molecular N—H⋯O hydrogen bonds. In the crystal, mol­ecules are connected by inter­molecular N—H⋯O hydrogen bonds into chains running along the *a* axis.

## Related literature

For background to acesulfame [systematic name: 6-methyl-1,2,3-oxathia­zin-4(3*H*)-one 2,2-dioxide], see: Clauss & Jensen (1973 ▶); Duffy & Anderson (1998 ▶); O’Brien Nabors (2001 ▶); İçbudak *et al.* (2006 ▶) For the crystal structures of acesulfame and its metal complexes, see: Beck *et al.* (1985 ▶); Bulut *et al.* (2005 ▶); Cavicchioli *et al.* (2010 ▶); İçbudak *et al.* (2005*a* ▶, 2006 ▶, 2007*b* ▶); Şahin *et al.* (2009 ▶, 2010 ▶); Velaga *et al.* (2010 ▶) and for spectroscopic, thermal analysis, magnetic susceptibility and conductivity studies on metal complexes of acesulfame, see: Beck *et al.* (1985 ▶); İçbudak *et al.* (2005*a* ▶,*b* ▶, 2006 ▶, 2007*a* ▶,*b* ▶). For Cu^2+^ complexes with an octa­hedral coordination geometry, see: Bulut *et al.* (2005 ▶); İçbudak *et al.* (2007*b* ▶); Pariya *et al.* (1998*a* ▶,*b* ▶); Şahin *et al.* (2010 ▶). For the Jahn–Teller effect, see: Jahn & Teller (1937 ▶). For the structural flexibility owing to the electronic configuration, see: Kozlevčar *et al.* (2006 ▶). For the octahedral geometry of the Cu^2+^ ion, see: Petric *et al.* (1998 ▶);
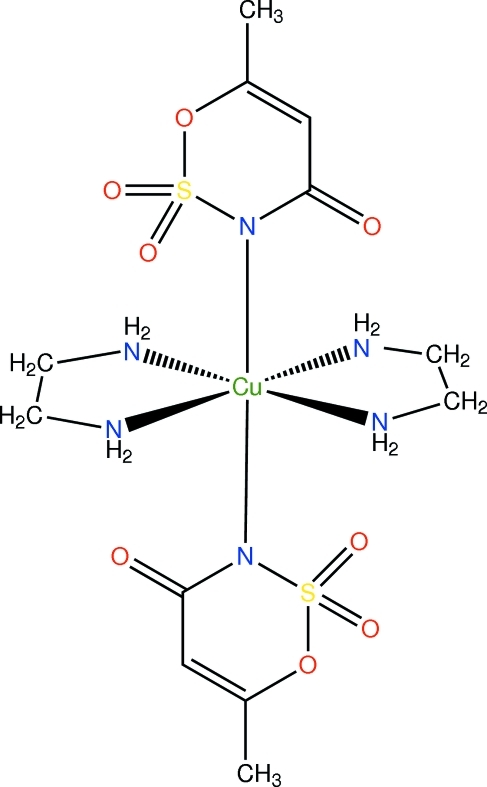


## Experimental

### 

#### Crystal data

[Cu(C_4_H_4_NO_4_S)_2_(C_2_H_8_N_2_)_2_]
*M* *_r_* = 508.03
Monoclinic, 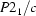

*a* = 6.9853 (3) Å
*b* = 17.5355 (6) Å
*c* = 8.4092 (4) Å
β = 93.017 (3)°
*V* = 1028.62 (7) Å^3^
*Z* = 2
Mo *K*α radiation
μ = 1.32 mm^−1^
*T* = 296 K
0.75 × 0.47 × 0.32 mm

#### Data collection

Stoe IPDS 2 diffractometer
Absorption correction: integration (*X-RED32*; Stoe & Cie, 2002 ▶) *T*_min_ = 0.438, *T*_max_ = 0.678
14620 measured reflections
2023 independent reflections
1865 reflections with *I* > 2σ(*I*)
*R*_int_ = 0.036

#### Refinement

*R*[*F* ^2^ > 2σ(*F* ^2^)] = 0.024
*wR*(*F* ^2^) = 0.065
*S* = 1.09
2023 reflections
150 parameters
H atoms treated by a mixture of independent and constrained refinement
Δρ_max_ = 0.23 e Å^−3^
Δρ_min_ = −0.25 e Å^−3^

### 

Data collection: *X-AREA* (Stoe & Cie, 2002 ▶); cell refinement: *X-AREA*; data reduction: *X-RED* (Stoe & Cie, 2002 ▶); program(s) used to solve structure: *WinGX* (Farrugia, 1997 ▶) and *SHELXS97* (Sheldrick, 2008 ▶); program(s) used to refine structure: *SHELXL97* (Sheldrick, 2008 ▶); molecular graphics: *ORTEP-3 for Windows* (Farrugia, 1997 ▶) and *Mercury* (Macrae *et al.*, 2006 ▶); software used to prepare material for publication: *WinGX* (Farrugia, 1999 ▶) and *PLATON* (Spek, 2009 ▶).

## Supplementary Material

Crystal structure: contains datablock(s) I, global. DOI: 10.1107/S1600536811051658/bt5731sup1.cif

Structure factors: contains datablock(s) I. DOI: 10.1107/S1600536811051658/bt5731Isup2.hkl

Additional supplementary materials:  crystallographic information; 3D view; checkCIF report

## Figures and Tables

**Table 1:** Selected bond lengths (Å)

Cu1—N1	2.7434 (15)
Cu1—N2	2.0090 (16)
Cu1—N3	2.0101 (14)

**Table 2:** Hydrogen-bond geometry (Å, °)

*D*—H⋯*A*	*D*—H	H⋯*A*	*D*⋯*A*	*D*—H⋯*A*
N3—H3*B*⋯O4	0.82 (2)	2.20 (2)	2.905 (2)	143 (2)
N2—H2*A*⋯O4^i^	0.82 (2)	2.13 (2)	2.931 (2)	167 (2)
N2—H2*B*⋯O1^ii^	0.87 (2)	2.57 (2)	3.250 (2)	137 (2)
N3—H3*A*⋯O2^iii^	0.85 (2)	2.23 (2)	2.974 (2)	147 (2)

Symmetry codes: (i) 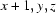; (ii) 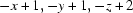; (iii) 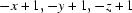.

**Fig. 1.**
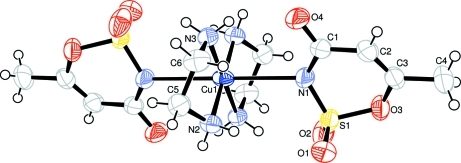
The molecular structure of the title compound, showing the atomic numbering scheme. Displacement ellipsoids are drawn at the 50% probability level.

**Fig. 2.**
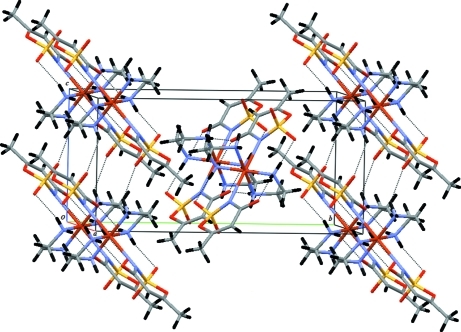
The two-dimensional sheet structure of the title compound with the hydrogen bonds.

## supplementary crystallographic information

### Comment

Clauss and Jensen discovered acesulfame   (Clauss & Jensen, 1973). Acesulfame (acs), (C_4_H_5_SO_4_N), is systematically named 6-methyl-1,2,3-oxathiazin-4(3*H*)-one 2,2-dioxide and an oxathiazinone dioxide. It, meanwhile, is known as 6-methyl-3,4-dihydro-1,2,3-oxathiazin-4-one 2,2-dioxide or acetosulfam. Acesulfame has been widely used as a non-caloric artificial sweetener (Duffy *et al.* 1998; O'Brien Nabors, 2001) since 1988, after the FDA (US Food and Drug Administration) granted approval. Acesulfame which is nonnutritive sweetener have been widely used by the food industries to replace sugars in foods. Acesulfame is without calories and it is not digested, accumulated and changed in the human metabolism and quickly excreted from the body (Duffy *et al.*, 1998; O'Brien Nabors, 2001). Furthermore, acesulfam K can withstand high cooking temperature (Duffy *et al.*, 1998). The artificial sweetener acesulfame has not only biological importance but also interest chemical properties. Because acesulfame ion (acs) has potential donor atoms as the imino nitrogen, ring oxygen, one carbonyl and two sulfonyl O atoms, which can be utilized in forming coordination bonds with different metal ions (İçbudak *et al.*, 2006).

The crystal structures of acesulfame and its metal complexes have been reported previously (Beck *et al.*, 1985; Bulut *et al.*, 2005; Cavicchioli *et al.*, 2010; İçbudak *et al.*, 2005*a*; İçbudak *et al.*, 2006; İçbudak *et al.*, 2007*b*; Şahin *et al.*, 2009; Şahin *et al.*, 2010; Velaga *et al.*, 2010). Furthermore, NMR (Beck *et al.*, 1985), electronic (İçbudak *et al.*, 2006; İçbudak *et al.*, 2007*a*; İçbudak *et al.*, 2007*b*), IR (Beck *et al.*, 1985; İçbudak *et al.*, 2006; İçbudak *et al.*, 2007*a*; İçbudak *et al.*, 2007*b*), mass (İçbudak *et al.* 2005*b*) spectroscopies, thermal analysis (İçbudak *et al.*, 2005*b*; İçbudak *et al.*, 2006; İçbudak *et al.*, 2007*a*; İçbudak *et al.*, 2007*b*), magnetic susceptibility (İçbudak *et al.*, 2006; İçbudak *et al.*, 2007*a*), conductivity (İçbudak *et al.*, 2007*b*) studies have been performed on the metal complexes of acesulme. In addition, the stability belong to different form of the acesulfame have being studied (Velaga *et al.*, 2010).

Here, we report *trans*-bis(acesulfamato-*N*)bis(ethylenediamine-N,*N*')copper(II) coplex as shown Fig. 1. In the crystal structure, Cu^2+^ ion is six-coordination by six N atoms from two ethylenediamine and two acesulfamato ligands in a octahedron coordination geometry. Similarly, the Cu^2+^ complexes in literature had been reported in octahedron coordination geometry (Bulut *et al.*, 2005; İçbudak *et al.*, 2007*b*; Pariya *et al.*, 1998*a*; Pariya *et al.*, 1998*b*; Şahin *et al.*, 2010). The bond distances between Cu^2+^ and N atoms for the Cu1—N1, Cu1—N2 and Cu1—N3 were found as 2.7432 (15) Å, 2.0091 (15) Å and 2.0103 (14) Å, respectively. As can be seen, the Cu1—N1 bond distance longer than the Cu1—N2 and Cu1—N3 bond distances and this is called as the Jahn-Teller effect (Jahn & Teller, 1937). Cu(II) with d^9^ electronic configuration is Jahn-Teller active in an octahedron coordination sphere. Because of odd d electron occupies one of the d-orbitals, crystal structure has structural flexibility (Kozlevčar *et al.*, 2006). The crystal structure reported by (Şahin *et al.*, 2010) is similarly the our crystal structure with Jahn-Teller effect. The bond distance between N atoms in axial positions and Cu^2+^ ion had been reported as 2.7175 (16) Å by (Şahin *et al.*, 2010). The bond distances of some crystal structures that has Jahn-Teller effect in literature are given in Table 3. Because of the N1—Cu1—N2 and N1—Cu1—N3 bond angles are 86.96 (5)° and 87.86 (5)°, repectively, the ethylenediamine group in equatorial plane and acesulfamato ligand axial position are almost perpendicular to each other. Some of the bond distances and bond angles belong to crystal structure are given in Table 1.

The title compound, *trans*-bis(acesulfamato-*N*)bis(ethylenediamine-N,*N*')copper(II), has intramolecular and intermolecular N—H···O hydrogen bonds. These hydrogen bonds play an important role for packing of molecules. The intra- and intermolecular hydrogen bonds are occurred *via* the O_carbonyl_ and O_suphonyl_ of acesulfamoto ligand which are donor atoms. Inramolecular N3—H3B···O4 and N3—H3A···O2^iii^ [symmetry code iii: -*x* + 1, -*y* + 1, -*z* + 1] hydrogen bonds are between the N atoms of ethylenediamine coordinated with the Cu^2+^ ion and the O_sulfonyl_ and O_carbonyl_ of acesulfamato ligand. The geometric parameters of these hydrogen bonds are 0.82 (2) Å, 2.21 (82) Å, 2.905 (2) Å, 144 (2)° and 0.87 (2) Å, 2.21 (2) Å, 2.973 (2) Å, 147.5 (19)°, respectively. The intermolecular hydrogen bonds are between the N atoms of ethylenediamine and the O_carbonyl_ and other O_sulfonyl_ atoms of acesulfamato ligand. The details of hydrogen bonds are shown in Table 2. In the crystal structure, the N2—H2B···O1 intermolecular hydrogen bonds along the [001] produce *R*_2_^2^(12) motifs. Similarly, the N2—H2A···O4 intermolecular hydrogen bonds along the [100] create *R*_2_^2^(12) ring motifs. The two-dimensional sheet are produced by the combination of the N—H···O intermolecular hydrogen bonds at the *ac-*plane as shown Fig. 2.

### Experimental

The complex was prepared upon treatment of 1 mmol [Cu(acs)_2_(H_2_O)] in 50 ml e thanol with 2 mmol e thylenediamine in 50 ml e thanol by stirring for 2 h at 50 C temperature. After cooling the reaction mixture to room temperature, the formed violet crystals were filtered, washed with alcohol and acetone, and dried in vacuum.

### Refinement

H atoms attached to C atoms were positioned geometrically [C—H=0.930, 0.960 or 0.970 Å] and treated as riding with *U*_iso_(H)=1.2*U*_eq_(C) and 1.5*U*_eq_(C). Other H atoms were located in a difference map and refined freely.

### Crystal data

**Table:**

[Cu(C_4_H_4_NO_4_S)_2_(C_2_H_8_N_2_)_2_]	*F*(000) = 526
*M**_r_* = 508.03	*D*_x_ = 1.640 Mg m^−^^3^
Monoclinic, *P*2_1_/*c*	Mo *K*α radiation, λ = 0.71073 Å
Hall symbol: -P 2ybc	Cell parameters from 28030 reflections
*a* = 6.9853 (3) Å	θ = 2.3–28.0°
*b* = 17.5355 (6) Å	µ = 1.32 mm^−^^1^
*c* = 8.4092 (4) Å	*T* = 296 K
β = 93.017 (3)°	Prism, violet
*V* = 1028.62 (7) Å^3^	0.75 × 0.47 × 0.32 mm
*Z* = 2	

### Data collection

**Table:**

Stoe IPDS 2 diffractometer	2023 independent reflections
Radiation source: fine-focus sealed tube	1865 reflections with *I* > 2σ(*I*)
graphite	*R*_int_ = 0.036
w–scan rotation	θ_max_ = 26.0°, θ_min_ = 2.3°
Absorption correction: integration (*X-RED32*; Stoe & Cie, 2002)	*h* = −8→8
*T*_min_ = 0.438, *T*_max_ = 0.678	*k* = −21→21
14620 measured reflections	*l* = −10→10

### Refinement

**Table:**

Refinement on *F*^2^	Primary atom site location: structure-invariant direct methods
Least-squares matrix: full	Secondary atom site location: difference Fourier map
*R*[*F*^2^ > 2σ(*F*^2^)] = 0.024	Hydrogen site location: inferred from neighbouring sites
*wR*(*F*^2^) = 0.065	H atoms treated by a mixture of independent and constrained refinement
*S* = 1.09	*w* = 1/[σ^2^(*F*_o_^2^) + (0.0309*P*)^2^ + 0.3289*P*] where *P* = (*F*_o_^2^ + 2*F*_c_^2^)/3
2023 reflections	(Δ/σ)_max_ = 0.001
150 parameters	Δρ_max_ = 0.23 e Å^−^^3^
0 restraints	Δρ_min_ = −0.25 e Å^−^^3^

### Special details

**Table:**

Geometry. All e.s.d.'s (except the e.s.d. in the dihedral angle between two l.s. planes) are estimated using the full covariance matrix. The cell e.s.d.'s are taken into account individually in the estimation of e.s.d.'s in distances, angles and torsion angles; correlations between e.s.d.'s in cell parameters are only used when they are defined by crystal symmetry. An approximate (isotropic) treatment of cell e.s.d.'s is used for estimating e.s.d.'s involving l.s. planes.
Refinement. Refinement of *F*^2^ against ALL reflections. The weighted *R*-factor *wR* and goodness of fit *S* are based on *F*^2^, conventional *R*-factors *R* are based on *F*, with *F* set to zero for negative *F*^2^. The threshold expression of *F*^2^ > σ(*F*^2^) is used only for calculating *R*-factors(gt) *etc*. and is not relevant to the choice of reflections for refinement. *R*-factors based on *F*^2^ are statistically about twice as large as those based on *F*, and *R*- factors based on ALL data will be even larger.

### Fractional atomic coordinates and isotropic or equivalent isotropic displacement parameters (Å^2^)

**Table:**

	*x*	*y*	*z*	*U*_iso_*/*U*_eq_	
C1	0.1200 (3)	0.55390 (12)	0.7619 (2)	0.0473 (4)	
C2	0.0228 (3)	0.59733 (13)	0.8823 (2)	0.0526 (5)	
H2	−0.0916	0.5781	0.9177	0.063*	
C3	0.0877 (3)	0.66195 (12)	0.9434 (2)	0.0483 (4)	
C4	−0.0106 (3)	0.71462 (15)	1.0516 (3)	0.0697 (6)	
H4A	−0.1428	0.7001	1.0557	0.105*	
H4B	−0.0027	0.7659	1.0123	0.105*	
H4C	0.0502	0.7118	1.1565	0.105*	
C5	0.5868 (3)	0.36713 (11)	0.6731 (2)	0.0508 (5)	
H5A	0.6284	0.3403	0.7697	0.061*	
H5B	0.6471	0.3438	0.5839	0.061*	
C6	0.3715 (3)	0.36387 (11)	0.6486 (3)	0.0539 (5)	
H6A	0.3310	0.3119	0.6257	0.065*	
H6B	0.3123	0.3805	0.7444	0.065*	
Cu1	0.5000	0.5000	0.5000	0.03988 (11)	
N1	0.2955 (2)	0.57689 (9)	0.71898 (19)	0.0463 (4)	
N2	0.6387 (2)	0.44831 (9)	0.6856 (2)	0.0435 (3)	
N3	0.3116 (2)	0.41394 (9)	0.51456 (19)	0.0404 (3)	
O1	0.5067 (2)	0.59346 (10)	0.96415 (19)	0.0653 (4)	
O2	0.5347 (2)	0.68003 (9)	0.7441 (2)	0.0699 (5)	
O3	0.26382 (19)	0.69080 (8)	0.90616 (17)	0.0539 (3)	
O4	0.0388 (2)	0.49923 (9)	0.6937 (2)	0.0673 (4)	
S1	0.41531 (6)	0.63171 (3)	0.83179 (6)	0.04469 (13)	
H2A	0.755 (3)	0.4555 (13)	0.691 (3)	0.054 (6)*	
H2B	0.602 (3)	0.4650 (14)	0.776 (3)	0.055 (6)*	
H3A	0.307 (3)	0.3881 (13)	0.429 (3)	0.053 (6)*	
H3B	0.207 (3)	0.4339 (13)	0.526 (3)	0.056 (6)*	

### Atomic displacement parameters (Å^2^)

**Table:**

	*U*^11^	*U*^22^	*U*^33^	*U*^12^	*U*^13^	*U*^23^
C1	0.0385 (9)	0.0561 (11)	0.0471 (10)	−0.0003 (8)	0.0002 (8)	−0.0054 (8)
C2	0.0387 (9)	0.0678 (13)	0.0519 (11)	−0.0002 (9)	0.0087 (8)	−0.0065 (10)
C3	0.0449 (10)	0.0571 (11)	0.0431 (10)	0.0123 (9)	0.0050 (8)	0.0006 (8)
C4	0.0639 (14)	0.0801 (16)	0.0662 (13)	0.0215 (12)	0.0136 (11)	−0.0153 (12)
C5	0.0590 (12)	0.0398 (9)	0.0528 (11)	0.0035 (8)	−0.0063 (9)	0.0067 (8)
C6	0.0591 (12)	0.0445 (10)	0.0572 (12)	−0.0135 (9)	−0.0039 (9)	0.0123 (9)
Cu1	0.03489 (16)	0.03563 (17)	0.04820 (19)	−0.00665 (12)	−0.00646 (12)	0.00794 (12)
N1	0.0406 (8)	0.0500 (8)	0.0490 (8)	−0.0035 (7)	0.0078 (6)	−0.0126 (7)
N2	0.0373 (8)	0.0447 (8)	0.0481 (9)	−0.0025 (7)	−0.0033 (7)	0.0033 (7)
N3	0.0376 (8)	0.0395 (8)	0.0440 (9)	−0.0044 (6)	0.0015 (6)	−0.0016 (7)
O1	0.0569 (9)	0.0708 (10)	0.0666 (9)	0.0108 (7)	−0.0126 (7)	−0.0071 (8)
O2	0.0744 (10)	0.0541 (8)	0.0844 (11)	−0.0224 (8)	0.0346 (9)	−0.0203 (8)
O3	0.0539 (8)	0.0445 (7)	0.0645 (9)	0.0039 (6)	0.0136 (7)	−0.0125 (6)
O4	0.0424 (8)	0.0831 (11)	0.0769 (10)	−0.0151 (7)	0.0067 (7)	−0.0326 (9)
S1	0.0409 (2)	0.0421 (2)	0.0517 (3)	−0.00205 (18)	0.00779 (19)	−0.01057 (19)

### Geometric parameters (Å, °)

**Table:**

C1—O4	1.239 (2)	C6—H6B	0.9700
C1—N1	1.357 (2)	Cu1—N1	2.7434 (15)
C1—C2	1.462 (3)	Cu1—N1^i^	2.7434 (15)
C2—C3	1.315 (3)	Cu1—N2	2.0090 (16)
C2—H2	0.9300	Cu1—N2^i^	2.0090 (16)
C3—O3	1.381 (2)	Cu1—N3	2.0101 (14)
C3—C4	1.489 (3)	Cu1—N3^i^	2.0102 (14)
C4—H4A	0.9600	N1—S1	1.5624 (15)
C4—H4B	0.9600	N2—H2A	0.82 (2)
C4—H4C	0.9600	N2—H2B	0.87 (2)
C5—N2	1.471 (2)	N3—H3A	0.85 (2)
C5—C6	1.509 (3)	N3—H3B	0.82 (2)
C5—H5A	0.9700	O1—S1	1.4218 (16)
C5—H5B	0.9700	O2—S1	1.4220 (15)
C6—N3	1.472 (2)	O3—S1	1.6292 (13)
C6—H6A	0.9700		
			
O4—C1—N1	120.27 (17)	N1—Cu1—N3	87.84 (5)
O4—C1—C2	120.39 (17)	N2—Cu1—N2^i^	180.0
N1—C1—C2	119.23 (17)	N2—Cu1—N3	84.51 (7)
C3—C2—C1	123.79 (18)	N2^i^—Cu1—N3	95.49 (7)
C3—C2—H2	118.1	N2—Cu1—N3^i^	95.49 (7)
C1—C2—H2	118.1	N2^i^—Cu1—N3^i^	84.51 (7)
C2—C3—O3	121.33 (17)	N3—Cu1—N3^i^	180.00 (6)
C2—C3—C4	127.8 (2)	C1—N1—S1	118.93 (13)
O3—C3—C4	110.88 (19)	C5—N2—Cu1	105.92 (12)
C3—C4—H4A	109.5	C5—N2—H2A	113.1 (16)
C3—C4—H4B	109.5	Cu1—N2—H2A	114.0 (16)
H4A—C4—H4B	109.5	C5—N2—H2B	107.8 (16)
C3—C4—H4C	109.5	Cu1—N2—H2B	112.0 (15)
H4A—C4—H4C	109.5	H2A—N2—H2B	104 (2)
H4B—C4—H4C	109.5	C6—N3—Cu1	109.52 (11)
N2—C5—C6	106.65 (15)	C6—N3—H3A	109.1 (15)
N2—C5—H5A	110.4	Cu1—N3—H3A	110.4 (15)
C6—C5—H5A	110.4	C6—N3—H3B	112.7 (16)
N2—C5—H5B	110.4	Cu1—N3—H3B	106.0 (16)
C6—C5—H5B	110.4	H3A—N3—H3B	109 (2)
H5A—C5—H5B	108.6	C3—O3—S1	117.28 (12)
N3—C6—C5	108.84 (15)	O1—S1—O2	115.84 (11)
N3—C6—H6A	109.9	O1—S1—N1	112.91 (10)
C5—C6—H6A	109.9	O2—S1—N1	111.20 (9)
N3—C6—H6B	109.9	O1—S1—O3	105.87 (9)
C5—C6—H6B	109.9	O2—S1—O3	103.33 (8)
H6A—C6—H6B	108.3	N1—S1—O3	106.64 (8)
N1—Cu1—N2	86.97 (6)		
			
O4—C1—C2—C3	−170.1 (2)	N2—Cu1—N3—C6	1.51 (14)
N1—C1—C2—C3	6.0 (3)	N2^i^—Cu1—N3—C6	−178.49 (14)
C1—C2—C3—O3	−5.2 (3)	C2—C3—O3—S1	−18.8 (2)
C1—C2—C3—C4	172.3 (2)	C4—C3—O3—S1	163.28 (15)
N2—C5—C6—N3	52.2 (2)	C1—N1—S1—O1	78.40 (17)
O4—C1—N1—S1	−165.20 (17)	C1—N1—S1—O2	−149.42 (16)
C2—C1—N1—S1	18.7 (3)	C1—N1—S1—O3	−37.46 (18)
C6—C5—N2—Cu1	−49.01 (18)	C3—O3—S1—O1	−82.80 (15)
N3—Cu1—N2—C5	26.67 (13)	C3—O3—S1—O2	154.99 (15)
N3^i^—Cu1—N2—C5	−153.33 (13)	C3—O3—S1—N1	37.68 (16)
C5—C6—N3—Cu1	−29.1 (2)		

Symmetry codes: (i) −*x*+1, −*y*+1, −*z*+1.

### Hydrogen-bond geometry (Å, °)

**Table:**

*D*—H···*A*	*D*—H	H···*A*	*D*···*A*	*D*—H···*A*
N3—H3B···O4	0.82 (2)	2.20 (2)	2.905 (2)	143 (2)
N2—H2A···O4^ii^	0.82 (2)	2.13 (2)	2.931 (2)	167 (2)
N2—H2B···O1^iii^	0.87 (2)	2.57 (2)	3.250 (2)	137 (2)
N3—H3A···O2^i^	0.85 (2)	2.23 (2)	2.974 (2)	147 (2)

Symmetry codes: (ii) *x*+1, *y*, *z*; (iii) −*x*+1, −*y*+1, −*z*+2; (i) −*x*+1, −*y*+1, −*z*+1.

### Table 3 Comparision Cu—N,O bond distance (Å) in related compounds

**Table:**

Compound	Cu—N, O (equatorial)	Cu—N, O (axial)	References
[Cu(C_4_H_4_NO_4_S)_2_(C_2_H_8_N_2_)_2_]	2.0092 (16), 2.0103 (15)	2.7434 (15)	This work
[Cu(C_4_H_4_NO_4_S)_2_(C_6_H_14_N_2_)_2_]	2.0077 (17), 2.0036 (18)	2.7175 (16)	Şahin *et al.* (2010)
[Cu(C_4_H_12_N_2_)_2_(H_2_O)_2_](C_4_H_4_NO_4_S)_2_'	2.035 (2), 2.051 (2)	1.245 (3), 2.479 (2)	İçbudak *et al.* (2007*b*)
[Cu(C_4_H_4_NO_4_S)_2_(C_4_H_5_N_3_)_2_]	2.0046 (16), 2.0107 (13)	2.4597 (16)	Bulut *et al.* (2005)
C_12_H_32_Cl_2_N_4_O_2_Cu	2.044 (2), 2.027 (3)	2.590 (2)	Pariya *et al.* (1998*a*)
C_12_H_28_N_6_O_6_Cu	1.990 (4), 2.006 (4)	2.620 (3)	Pariya *et al.*, 1998*a*
Cu(O_2_CC_8_H_17_)_2_(NH_2_C_2_H_4_OH)_2_	1.999 (3), 2.009 (2)	2.477 (2)	Petric *et al.* (1998)
